# Improvement of the Impact Resistance of Epoxy Prepregs Through the Incorporation of Polyamide Nonwoven Fabric

**DOI:** 10.3390/ma18030661

**Published:** 2025-02-02

**Authors:** Anastasia Kondrateva, Oleg Morozov, Erdni Erdni-Goryaev, Ekaterina Afanaseva, Viktor Avdeev

**Affiliations:** Department of Chemistry, M. V. Lomonosov Moscow State University, 119991 Moscow, Russia

**Keywords:** compression after impact, nonwoven fabric, Polyamide 12, epoxy prepreg, autoclave forming

## Abstract

The impact of introducing a nonwoven polyamide PA 12-E material on the mechanical properties of polymer composite materials based on epoxy autoclave prepreg T107 has been investigated. This study demonstrates that the incorporation of nonwoven fabric does not lead to a decrease in the mechanical properties of the composites. A significant advantage of composites reinforced with nonwoven fabric is their enhanced impact resistance. During a free impact with an energy of 6.67 J per 1 mm of the sample, complete breakdown with fiber destruction occurs in samples without nonwoven material. In contrast, samples containing nonwoven material exhibit damage characterized by stratification without compromising the fibers. The compressive strength after impact increased from 260 to 320 MPa with the addition of nonwoven material. Consequently, the proposed modification of the commercial prepreg will expand the material’s range of applications and enhance safety, particularly in aircraft structures.

## 1. Introduction

Trends in aerospace structures are focused on increasing the proportion of carbon-fiber-reinforced polymer (CFRP) materials in aircraft components [1,2,3]. This shift is primarily due to their high specific mechanical properties. The use of thermosetting resins, which are inherently brittle and possess relatively low strength, results in composites with low damage tolerance that are susceptible to accidental impact damage—such as from runway debris, bird strikes, or dropped tools during maintenance [4]. Impact damage leads to severe degradation of the load-bearing capacity of the structure due to matrix cracking, delamination, and fiber breakage. This degradation can be a limiting factor in the structural applications of composites as the strength of composite structures significantly decreases after an impact, with loaded fibers either breaking or no longer being adequately supported by the damaged matrix. However, a significant challenge in replacing metals with CFRP materials is their low impact resistance, which restricts their application [1,4,5].

Delamination is arguably the most critical damage mechanism in laminated composites as it significantly reduces residual compressive strength. The delamination phenomenon can be caused by the concentration of interlaminar stresses that occur in the vicinity of the free edges or in and around the holes in laminated plates [6]. In addition, the interlaminar defects can grow under compressive loading. In this case, the thin laminated layers degrade (deboning interfaces) and are responsible for increased stresses in the vicinity of the boundaries of delaminated surfaces. In the analysis of the delamination, the stage of the crack initiation can be distinguished from the phase of the crack propagation.

Consequently, enhancing and optimizing the properties of composites to resist and tolerate damage from out-of-plane loading, such as impact events, has emerged as a vital area of research. The strength of thermosetting resins can be improved by developing a three-phase composite that consists of fiber, matrix, and reinforcement [7,8]. Various strategies are employed to enhance impact characteristics [9,10,11], including the incorporation of carbon nanotubes [12,13,14], chopped and ground carbon fiber [15,16,17,18], polymer films [19,20,21], chopped polymer fibers [22], nonwoven additives based on thermoplastic polymers [23,24,25,26], etc.

The enhancement of impact characteristics in epoxy composite materials, without a significant reduction in thermal and mechanical properties, can primarily be achieved by incorporating engineering thermoplastics into the final structure of carbon-fiber-reinforced polymers (CFRPs) [27,28]. A nonwoven material composed of thermoplastic polymers is utilized to introduce a controlled interpenetrating thermoplastic mesh. Furthermore, thermoplastic nonwovens possess a substantial specific surface area, which enhances adhesion to the polymer matrix phase. The properties of the final CFRP are directly influenced by the surface density and chemical composition of the nonwoven material, as well as the type of reinforcing filler and the manufacturing method employed for the CFRP [29]. Additionally, various factors, such as the adhesion between the nonwoven material and the matrix; the solubility of the thermoplastic during curing at elevated temperatures in epoxy resin [30], followed by phase separation during the curing process; and the CFRP molding conditions, significantly impact the behavior of CFRP products under shock loads [31].

The primary mechanisms by which interlaminar veils resist delamination and crack growth are fiber bridging, fiber pull-out, and fiber breakage. The specific mechanism that occurs during fracture depends on the bond between the matrix and the fiber. When the interface between the fiber and the matrix is weak, the fiber is easily pulled out of the matrix. Fiber pull-out enhances resistance to crack propagation by consuming energy to break the bond between the fiber and the matrix, allowing the fiber to be extracted. Conversely, if the interface between the fiber and the matrix is strong, the fiber remains anchored, leading to fiber bridging. In this scenario, fibers with ends on either side of the crack help resist crack opening due to the tension in the fibers. Fiber bridging is regarded as one of the most effective mechanisms for reducing stress levels in the vicinity of a crack. Additionally, fiber breakage contributes to increased crack propagation energy through the plastic deformation of ductile thermoplastic fibers [10].

In [32], the impact resistance was enhanced by incorporating a nonwoven material made from polyphenylene sulfide into the structure of the carbon-fiber-reinforced polymer (CFRP). The study demonstrated that both the number of thermoplastic layers and the orientation of the reinforcing carbon tape significantly influence the energy absorption capacity. The sample featuring a nonwoven material based on polyphenylene sulfide, with a surface density of 10 g/m^2^ placed between each layer of carbon tape, exhibited the highest efficiency.

The incorporation of nonwoven fabric made from polyamide-6.6 into the composition of a low-temperature cure CFRP with varying surface densities (17 and 50 g/m^2^) [33] resulted in a significant enhancement of the impact properties of CFRP compared to CFRP without nonwoven fabric. However, an increase in the amount of nonwoven fabric led to a deterioration in the mechanical properties of the resulting CFRPs. Therefore, it can be concluded that the content of nonwoven fabric can be adjusted based on the intended application of the material and the loads it will encounter during use, in order to achieve the desired characteristics.

The assessment of the impact of incorporating thermoplastics into the epoxy matrix on the brittle properties of the composite is typically conducted through crack resistance tests. These tests evaluate the composite’s resistance to brittle fracture under shear, separation, or combined loading conditions. However, from a practical perspective, the properties following an impact are of greater interest as such tests more accurately simulate damage that may occur in real aviation incidents, such as a tool falling onto a wing, a collision with birds, or an encounter with other vehicles. Previous studies have described the effects of shock loading on the compressive strength of a CFRP made from the epoxy autoclave prepreg T107 [34]. The compressive strength of a composite sample, which is based on a unidirectional non-slip prepreg reinforced with Formosa 12K filler in a thermosetting epoxy matrix (T107), was measured at 169 MPa after an impact energy of 6.7 J per 1 mm.

To assess the impact load effect on the performance characteristics of a CFRP, the most effective method is the impact compression test. In the article [35], the introduction of aramid nonwoven material to the epoxy prepreg resulted in an increase in impact compression strength from 165 MPa (without nonwoven material) to 230 MPa (with a nonwoven short aramid fiber veil).

There is almost no research on the effects of nonwoven materials on the properties of CFRPs under compression after impact. This gap highlights the necessity of studying this parameter as it is highly relevant for practical applications. This study aims to enhance the impact resistance of high-temperature cure CFRPs based on epoxy prepreg by incorporating a polyamide nonwoven material. A series of mechanical tests were conducted on CFRPs with and without the addition of the nonwoven material. Additionally, the samples were evaluated for compressive strength after impact as this characteristic closely resembles the impact toughness under the actual operating conditions of the material. This material can be utilized to manufacture wind turbine blades and aircraft engine blades.

## 2. Materials and Methods

The autoclave epoxy prepreg T107, produced by ITECMA LLC (Moscow, Russia), is derived from the unidirectional carbon tape UMT49-12K-EP manufactured by UMATEX (Moscow, Russia). Epoxy resin was based on Bisphenol A diglycidyl ether (DGEBA), N,N,O-Triglycidyl-p-aminophenol (EPAPh), and 3,3`diaminodiphenylsulfone (DDS) as a curing agent. Carbon fiber has a tensile strength of 4900 MPa and a tensile modulus of 260 GPa.

The nonwoven material was produced from PA 12-E polyamide granules manufactured by ANID LLC (St. Petersburg, Russia). It has a surface density of 5–8 g/m^2^ and a fiber diameter ranging from 10 to 35 µm, utilizing the extrusion molding method at temperatures between 290 and 310 °C. The description of the technological process was provided earlier in the article [36].

Ultrasonic testing (UT) was conducted at 25 °C in accordance with standard procedures, utilizing a UDL-2M laser-ultrasonic flaw detector equipped with a PLU-6P-02 converter (Moscow, Russia). A longitudinal-transverse scanning scheme was employed for the testing, with a scanning speed of ≤50 mm/s, a step size of ≤4 mm, and a frequency range of 2.7–13.6 MHz.

Dynamic mechanical analysis (DMA) was conducted following standard procedures using a TA Instruments DMA Q800 dynamo-mechanical analyzer (New Castle, USA). The samples, measuring 55 × 5 × 2 mm, were scanned over a temperature range of 50–250 °C at a frequency of 1 Hz in a nitrogen atmosphere (flow rate: 100 mL/min) with a heating rate of 5 °C/min. The samples were cut at an angle of 0° relative to the reinforcement axis.

Differential scanning calorimetry (DSC) was conducted using a NETZSCH DSC 204 Phoenix instrument (Selb, Germany). For the measurement, a sample weighing between 10 and 20 mg was placed in an aluminum crucible. The weighing accuracy was 10^−5^ g. Measurements were performed relative to an empty crucible in an argon atmosphere, with a volumetric gas flow rate of 100 mL/min. The temperature range for the measurement was set from −50 to 350 °C, with a heating rate of 10 °C/min. The melting point was determined as the inflection point where the heat flow changed in the initial section of the DSC curve.

The apparent interlaminar shear strength (ILSS) was determined using a Tinius Olsen 50ST device (Horsham, PA, USA) at 25 °C, with six samples measuring 20 × 10 mm. The method involves loading a sample that is freely supported at two points and applying a constant rate of 1.0 mm/min at the midpoint between the supports until failure occurs due to interlaminar shear. The key parameter measured is the magnitude of the applied load.

The tensile strength of the CFRP was measured using the Instron 5985 testing machine (Glenview, IL, USA) at 25 °C. A specially prepared elongated sample was positioned in the grips of the device and subjected to a constant loading speed of 5 mm/min. The maximum load value was recorded just before the sample failed, along with an assessment of the failure mode.

The compressive strength of the CFRP and its compressive strength after impact were measured using the Instron 5985 device at 25 °C. The testing method involved subjecting CFRP samples to short-term compression at a constant deformation rate of 1 mm/min. Each sample was securely fastened in a fixture positioned between the plates of the tensile testing machine. The compressive strength was calculated as the ratio of the maximum load applied before failure to the initial cross-sectional area of the sample.

The shear characteristics of the samples were determined using the Iosipescu method. Samples with a V-shaped notch were fabricated through precision cutting on a CNC milling machine. The tests were conducted using a Tinius Olsen universal tensile testing machine (Horsham, PA, USA) from the ST series, model 300ST, with a loading plate movement speed of 2 mm/min at room temperature. Shear deformation was measured using two mutually perpendicular strain gauges adhered to the test area.

SEM studies were conducted using a Vega 3 microscope (Tescan) (Brno—Kohoutovice, Czech Republic) equipped with secondary and backscattered electron detectors, operating at a voltage of 20 kV. The CFRP samples were shaped into cylindrical forms and filled with epoxy resin, followed by grinding and polishing. Prior to imaging, the polished surfaces were coated with a thin layer of gold, measuring 10–20 nm in thickness.

## 3. Results

**Figure 1:**
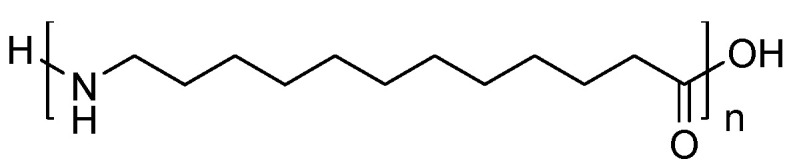
The structure of Polyamide 12.

Polyamide is particularly noteworthy as a thermoplastic toughening agent due to its ability to form covalent chemical bonds with an epoxy matrix. Additionally, both polyamide and epoxy resin are polar molecules, which enhances their compatibility and miscibility. This compatibility promotes interdiffusion, facilitating both mechanical and chemical crosslinking. Different types of polyamide (PA) are characterized by the number of carbon atoms in their monomers. A lower number of carbon atoms between amide groups results in the reactive amide groups being closer together, increasing the overall polarity and reactivity of the polymer [37,38]. Conversely, a longer hydrocarbon chain leads to reduced moisture absorption [39,40]. The softening point of Polyamide 12 is the lowest among all polyamides, which enhances the interaction between the epoxy matrix and the nonwoven material. Therefore, Polyamide 12 (Figure 1) was selected as the polymer for the production of nonwoven fabric.

**Figure 2:**
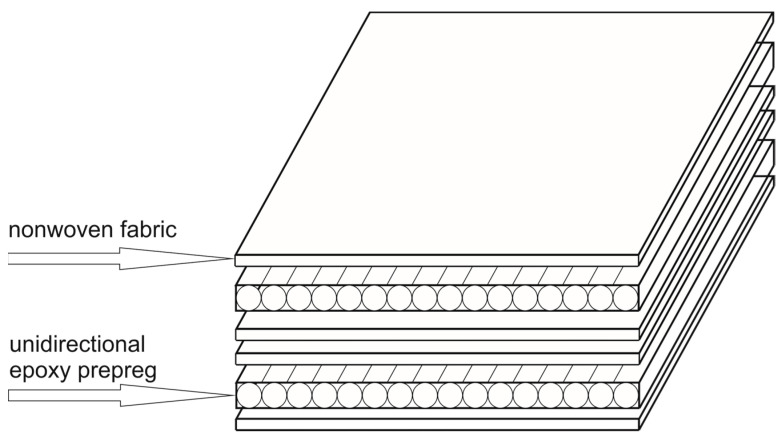
A scheme for arranging prepreg layers with alternating nonwoven materials is illustrated using the example of two layers for the production of the CFRP when laid out at [0]_10_.

To assess the influence of nonwoven materials on the properties of the carbon-fiber-reinforced polymer (CFRP), samples were fabricated using autoclave epoxy prepreg T107 (produced by ITECMA LLC, Moscow, Russia), which is derived from the unidirectional carbon tape UMT49-12K-EP manufactured by UMATEX (Moscow, Russia). Additionally, samples were prepared by incorporating a nonwoven material developed by Lomonosov Moscow State University [36]. This nonwoven material was applied to both sides of each prepreg layer (Figure 2).

**Figure 3:**
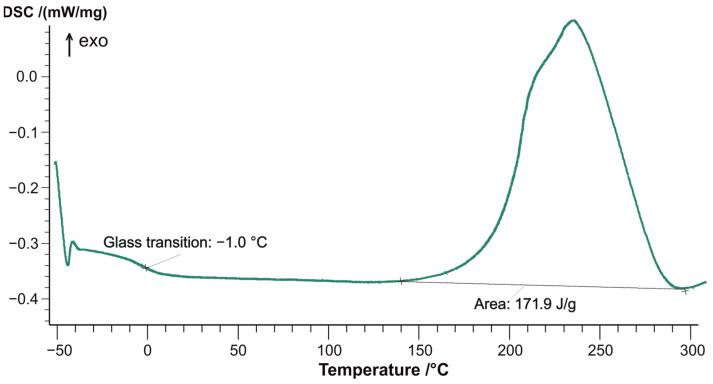
DSC curve of non-cured epoxy prepreg T107.

**Figure 4:**
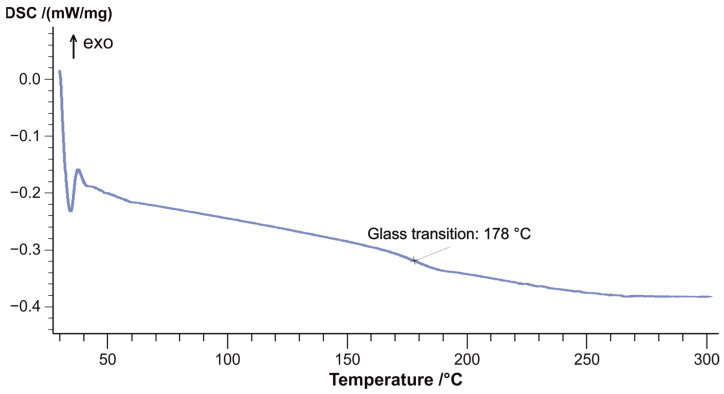
DSC curve of the cured CFRP sample using epoxy prepreg T107.

To determine the degree of curing of the epoxy matrix, the curing enthalpy of the T107 epoxy prepreg (Figure 3) was measured using the differential scanning calorimetry (DSC) method. As shown in Figure 4, the resin for sample T107 is fully cured, exhibiting a glass transition temperature of 178 °C.

**Figure 5:**
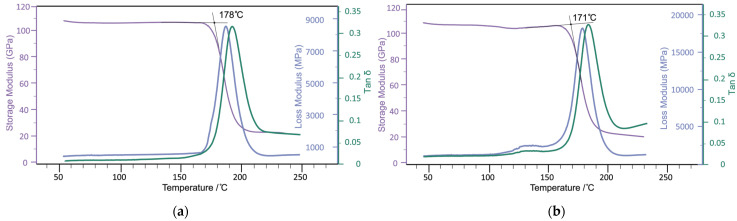
DMA curves of CFRP samples using epoxy prepreg T107 (**a**) and epoxy prepreg T107 with the addition of polyamide nonwoven material (**b**) are presented.

**Figure 6:**
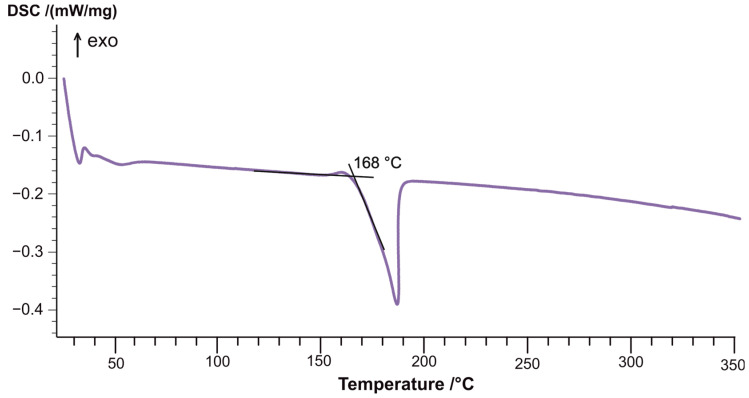
DSC curve of the polyamide sample PA 12-E.

To conduct mechanical tests and determine the glass transition temperature, samples with a [0]_10_ layout were fabricated using autoclave molding. As illustrated by the dynamic mechanical analysis (DMA) curves, the glass transition temperature of the CFRP sample based on the T107 prepreg is 178 °C too (Figure 5a) while the glass transition temperature of the CFRP sample containing nonwoven polyamide material is 171 °C (Figure 5b). This difference can be attributed to the melting onset temperature of the PA 12-E polyamide, which, according to the differential scanning calorimetry (DSC) curve (Figure 6), is 168 °C. Given that the operating temperature of the T107 prepreg does not exceed 120 °C, this reduction in the glass transition temperature does not adversely affect the performance characteristics of the final material.

Porosity, delamination, and cracks significantly impact the mechanical properties of composites and serve as critical points for defect propagation under impact loads. Therefore, to ensure an accurate comparison of material properties, it is essential to verify that the composites are free of defects prior to testing. The quality of the samples was assessed using a non-destructive ultrasonic method known as the echo method. For each sample, a strong bottom signal was obtained across the entire surface of the plate, indicating the integrity of the materials and the absence of defects.

The effect of polyamide nonwoven material on the impact properties of composites was evaluated by examining their resistance to damage when subjected to a falling load. To achieve this, samples of carbon-fiber-reinforced polymer (CFRP) plates with a [(+45/−45)(0/90)]6 s fiber orientation and a thickness of 5 mm were fabricated using the autoclave molding method. The samples were tested for impact resistance with a consistent energy level, normalized to the sample thickness, which was calculated based on the mass of the striker and the height from which the load was dropped. For the impact tests, a striker weighing 2.98 kg, featuring a smooth hemispherical tip with a diameter of 16 mm, was employed. To determine the numerical value of the effect of nonwoven material on the ability to withstand loads after impact, the ultimate compressive strength of the carbon-fiber-reinforced polymer (CFRP) post-impact was measured at 6.67 J/mm at a temperature of 25 °C.

It has been previously noted [41] that an increase in polyamide content leads to an enhancement in compressive strength following impact; however, the apparent strength significantly decreases in conjunction with interlaminar shear strength. The same trend is observed with the addition of one or two layers of nonwoven fabric, with the monolayer exhibiting growth. Based on the results presented in Table 1, the inclusion of two layers of nonwoven fabric was selected to achieve optimal properties of the CFRP.

The data obtained indicated that the addition of nonwoven material significantly increases the compressive strength after impact. However, this enhancement is accompanied by a decrease in interlaminar shear strength. As the content of nonwoven material continues to rise, there will be a substantial increase in the thickness of the monolayer and the weight of the CFRP, along with a further reduction in interlaminar shear strength. Therefore, two layers were selected to optimize the characteristics of the CFRP.

A series of tests were conducted to evaluate the effect of nonwoven material on the mechanical properties of the CFRP (see Table 2). Each test was performed on a set of seven samples. The results indicated that the incorporation of nonwoven material into the CFRP not only maintained the mechanical properties but also enhanced the existing values. The tensile strength test revealed a 7% increase in the measured indicators. The apparent ultimate strength at shear and the ultimate compressive strength of the CFRP remained consistent within the margin of error of the obtained values.

An exception is the test designed to determine the interlaminar shear strength of the CFRP. The retention of properties was 92% of the original T107. This can be attributed to the fact that during testing, failure occurs along the nonwoven material, which acts as a defect within the matrix.

**Figure 7:**
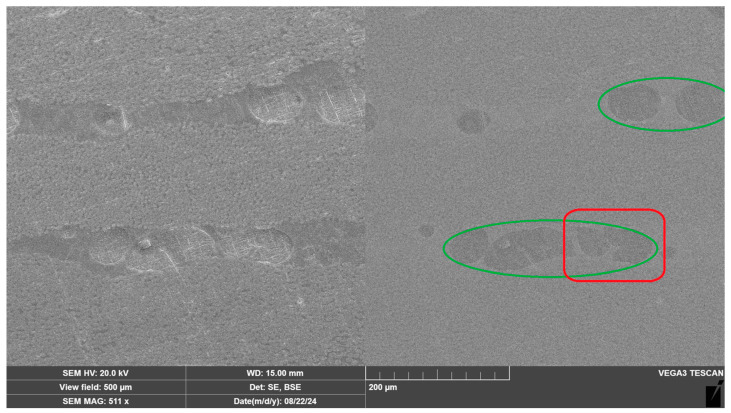
Scanning electron microscope (SEM) image of the terminal section of the CFRP incorporating nonwoven material, captured using secondary electron detection (**left**) and backscattered electron detection (**right**).

The thermoplastic phase distribution in the CFRP was evaluated using scanning electron microscopy. The image in Figure 7 shows the terminal section of the T107-based CFRP with nonwoven material. The secondary electron image (left) reveals the separation of the fiber and matrix phases; however, it is not possible to clearly identify the phase of the thermoplastic nonwoven material. Figure 7 (right) presents the same fragment of the CFRP but captured using backscattered electrons. Due to the differences in densities, imaging with backscattered electrons allows for the observation of thermoplastic inclusions (circled in green), indicating a uniform distribution between the layers of carbon fabric.

**Figure 8:**
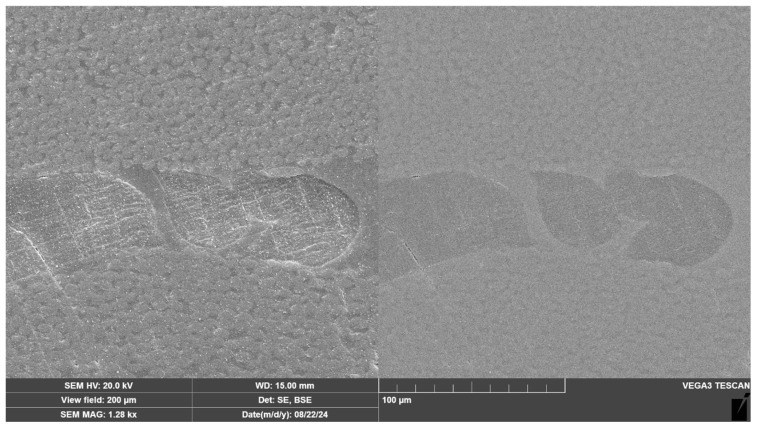
Scanning electron microscope (SEM) image illustrating the interaction between the nonwoven material with the CFRP matrix with secondary electron detection (**left**) and backscattered electron detection (**right**).

Figure 8 illustrates the area highlighted in red in Figure 7. Upon closer inspection, it is clear that the surface of the nonwoven material is thoroughly wetted with the binder, demonstrating a strong adhesion of the epoxy binder to the polyamide. The disordered orientation of the nonwoven material threads is also apparent as the cut is not only circular but also oval. This suggests that the fibers are oriented not only perpendicular to the cut but also at various angles relative to the reinforcement direction.

It is also evident that the structure of the nonwoven material is preserved despite melting at the molding and curing temperatures of the prepreg, which enhances the interaction between the matrix and Polyamide 12.

## 4. Conclusions

To enhance load resistance after impact, the epoxy prepreg was modified with a Polyamide 12 nonwoven fabric. The quantity of nonwoven fabric was carefully selected and its effect on the performance characteristics of the carbon-fiber-reinforced polymer was evaluated. The inclusion of the nonwoven fabric resulted in a slight reduction of the glass transition temperature to 171 °C; however, this decrease does not compromise the material’s usability at a maximum operating temperature of 120 °C. Electron microscopy analysis of the composite sections revealed a uniform distribution of the thermoplastic phase between the layers of carbon unidirectional tape, as well as strong adhesion between the polyamide and the epoxy matrix. While maintaining the mechanical properties, it was possible to increase the compressive strength after impact by 23%. Specifically, the compressive strength of the T107 epoxy prepreg sample was measured at 260 ± 5.2 MPa, whereas the nonwoven sample exhibited a compressive strength of 320 ± 6.6 MPa.

## Figures and Tables

**Table 1:** The impact of the number of layers of nonwoven material on the mechanical properties and specific characteristics of the CFRP at 25 °C.

Test Type	Regulatory Documentation	T107	T107 with One Layer of Nonwoven Material	T107 with Two Layers of Nonwoven Material
Compressive strength after impact σ_11_^−^, MPa	ASTM D7137 [42]	260 ± 5.2	290 ± 5.6	320 ± 6.6
Interlaminar shear strength τ_13_, MPa	ASTM D2344 [43]	106 ± 3.7	100 ± 1.5	97 ± 2.3
Prepreg surface density, g/m^2^	ISO 9864 [44]	308 ± 5.1	315 ± 10.3	328 ± 13.6
Monolayer thickness, mm	–	0.2 ± 0.03	0.21 ± 0.03	0.22 ± 0.04

**Table 2:** Results of the mechanical properties of the CFRP at 25 °C based on epoxy prepreg T107 and prepreg T107 with the addition of polyamide nonwoven material.

Test Type	Regulatory Documentation	T107	T107 with Nonwoven Material
Tensile strength σ_11_^+^, MPa	ASTM D3039 [45]	2136 ± 31.5	2300 ± 51.0
Tensile modulus Ε_11_^+^, GPa	ASTM D3039 [45]	139 ± 3.2	141 ± 2.9
Compressive strength σ_11_^−^, MPa	ASTM D 6641 [46]	957 ± 21.2	1264 ± 25.8
Interlaminar shear strength τ_13_, MPa	ASTM D2344 [43]	106 ± 3.7	97 ± 2.3
Shear strength τ_12_, MPa	ASTM D5379 [47]	147 ± 4.1	143 ± 3.8

## Data Availability

The original contributions presented in this study are included in the article. Further inquiries can be directed to the corresponding author.
